# Establishment of a Chinese street rabies virus library and its application for detecting neutralizing activity

**DOI:** 10.1186/s40249-018-0500-x

**Published:** 2018-12-05

**Authors:** Peng-Cheng Yu, Xiao-Yan Tao, Li-Hua Wang, Qing Tang, Li-Yun Fan, Shu-Xia Zhang, Shu-Qing Liu, Xue-Xin Lu, Gui-Zhen Wu, Wu-Yang Zhu

**Affiliations:** Key Laboratory of Medical Virology, Ministry of Health, National Institute for Viral Disease Control and Prevention, Chinese Center for Disease Control and Prevention, Beijing, 102206 China

**Keywords:** Street RABV, MAbs, Neutralizing activity

## Abstract

**Background:**

The injection of rabies immune globulin (RIG) is of the utmost importance in the management of category III exposures to rabies-suspect animals. Because of the high cost and limited availability of existing RIG, one possible replacement for RIG is monoclonal antibodies (MAbs) against the rabies virus (RABV). Consequently, it is necessary to determine the neutralizing activity of the MAbs against rabies viruses, especially street rabies virus. However, the method to detect the neutralizing activity of MAbs against street rabies virus remains undefined.

**Methods:**

To establish a method for detecting the neutralizing activity of MAbs against street rabies virus, we constructed a library consisting of 12 strains of street RABV from 11 provinces in China. Using this street RABV library and the Reed–Muench formula, we established a method for detecting the neutralizing titer of the MAbs. The reliability and repeatability of the method were evaluated by repeatedly measuring the neutralizing activity of a MAb and a post vaccination serum.

**Results:**

A total of 12 strains of street RABV were chosen for inclusion in the street RABV library, which covered six Chinese lineages (China I–China VI) and grew to high titers in N2A cells (> 10^5^ FFD_50_/ml). On the basis of the library, we constructed the method to detect the neutralizing activity of the MAbs. The results of repeatedly measuring the MAbs and positive serum showed excellent reliability and repeatability of the method established in this study.

**Conclusions:**

This study established a street RABV library reflecting the epidemiological features of Chinese rabies viruses, which provides a platform for detecting the neutralizing activity of MAbs against rabies viruses circulating in China.

**Electronic supplementary material:**

The online version of this article (10.1186/s40249-018-0500-x) contains supplementary material, which is available to authorized users.

## Multilingual abstracts

Please see Additional file 1 for translations of the abstract into the five official working languages of the United Nations.

## Background

Rabies is a fatal disease that is preventable when post exposure prophylaxis (PEP) is administered in a timely fashion. However, approximately 59 000 humans die from rabies each year [1]. The World Health Organization (WHO) recommends that prophylaxis for the prevention of disease in humans exposed to the rabies virus (RABV), which is a negative-sense single-stranded RNA (ssRNA) virus from the genus *Lyssavirus*, family Rhabdoviridae*,* should include prompt and thorough wound cleansing followed by passive vaccination with rabies immune globulin (RIG) and vaccination with the rabies vaccine. The report of the WHO/Bill and Melinda Gates Foundation Consultation agreed that the injection of rabies immune globulin into or around wounds is of the utmost importance in the management of category III exposures, which include single or multiple transdermal bites or scratches, licks on broken skin, contamination of mucous membranes with saliva from licks and exposure to bats. Rabies immunoglobulin neutralizes the rabies virus at the wound site during the period before the immune system responds to the vaccine via the production of RABV-neutralizing antibodies. Both active and passive immunization prevent the RABV from infiltrating the central nervous system but become ineffective once the virus has entered the central nervous system. Currently available rabies PEP for use in humans includes human rabies immune globulin (HRIG) and equine rabies immune globulin (ERIG). Treatment failures have occurred when RIG is delayed and not injected into the wound, if some deviation is made from the recommended PEP protocol, or when less than the recommended amount of RIG is administered [2, 3]. In addition, unpurified equine antirabies serum may still be used in some countries because of the shortage and high cost of HRIG and ERIG. The use of this antirabies serum is associated with higher rates of serious adverse reactions, including anaphylaxis [4]. Thus, because of the high costs and limited availability of existing RIGs, the WHO strongly recommends their replacement with a product that is at least equally potent and safer [5].

One possible replacement for RIG is monoclonal antibodies (MAbs) against the rabies virus. However, it is necessary to determine the neutralizing activity of each MAb against rabies viruses, especially street rabies virus, and compare it with that of HRIG. In our study, we constructed a library of Chinese street RABV strains including RABVs from different provinces, different hosts and different lineages, which was used to evaluate the neutralizing activity of MAbs against different street RABVs.

## Methods

### Street RABV, HRIG, neuro-2a cells, MAbs and serum

Table 1 lists the 24 strains of the rabies virus from 14 different provinces that were selected to establish the Chinese street RABV library. This library included street RABVs from four different hosts, including six samples from human brain tissue, two from cow brain tissue, one from ferret-badger brain tissue, and 15 from canine brain tissue.

**Table 1:** Street rabies virus strains used in this study

Code	Host	Province	Code	Host	Province
CHN0610H	Human	Hunan	CNX8601	Human	Ningxia
CHN0633D	Dog	Hunan	CNX8511	Human	Ningxia
CHN0635H	Human	Hunan	GDZQ45	Dog	Guangdong
CYN1009D	Dog	Yunnan	GDZQ46	Dog	Guangdong
CYN1003C	Cow	Yunnan	CSC1015D	Dog	Sichuan
CYN1025H	Human	Yunnan	CSC1016D	Dog	Sichuan
CJX0903D	Dog	Jiangxi	CSX0904D	Dog	Shanxi
CJS0621D	Dog	Jiangsu	CSX0901D	Dog	Shanxi
CJS0840H	Human	Jiangsu	GuangxiCx14	Dog	Guangxi
CJS0847D	Dog	Jiangsu	CAH0501D	Dog	Anhui
CSD0710D	Dog	Shandong	D19	Ferret badger	Zhejiang
CQH1202D	Cow	Inner Mongolia	CNM1103C	Dog	Qinghai

The national reference standard HRIG (30 IU/ml) was purchased from the National Institute for Biological Standards and Control (Potters Bar, Herts, UK). The rabies direct fluorescent antibody (DFA) reagent (fluorescein isothiocyanate-conjugated antirabies nucleoprotein antibody) was purchased from Fujirebio Diagnostics, Inc. (Malvern, PA, US). The mouse neuroblast cell line Neuro-2a (CCL-131, obtained from the American Type Culture Collection, Manassas, VA, US), was used for the routine diagnosis of rabies. BSR cells (a cloned baby hamster kidney cell line) were cultured at 37 °C in a 5% CO_2_ humidified incubator and maintained in Dulbecco’s minimum essential medium (Gibco, Waltham, MA, USA). The standard challenge virus (CVS-11) was provided by the National Institutes for Food and Drug Control, China. TRN006, a human MAb against the rabies virus, which has been patented (CN201310007039.1), was supplied by Tsinghua University. Post vaccination serum used to investigate the repeatability was stored in our lab.

### Phylogenetic analysis of the 24 RABV strains

The glycoprotein gene of the 24 RABV strains was already sequenced by our lab [6, 7], and based on the complete G gene sequences of these strains and other representative strains from China, a phylogenetic tree was constructed using MEGA 7 software with the neighbor-joining (NJ) method and 1 000 bootstrap replicates [8].

### Titration of street RABV

All brain tissue suspensions (*w*/*v* = 30%) were homogenized in Modified Eagle Medium (MEM) (Sigma-Aldrich, St. Louis, MO, US) and then serially diluted 10-fold from 10^− 1^ to 10^− 8^ in MEM containing 10% fetal bovine serum (Gibco, Waltham, MA, US). Then, 100 μl of each dilution of the virus suspension was transferred into six replicate wells of a 96-well plate and incubated at 37 °C for 1 h. Next, 100 μl Neuro-2a cell suspension (5 × 10^5^ cells/ml) was added to each well, and the plates were incubated at 37 °C in 5% CO_2_ for 48 h. The DFA test was then performed to enumerate the foci of infection from street RABV and to calculate the titer of street RABV (50% fluorescent focus-forming dose per ml; FFD_50_/ml).

### DFA test

The 96-well plates were fixed in cold acetone, incubated with 50 μl/well of rabies DFA reagent at 37 °C for 30 min, then washed three times using 100 μl of phosphate-buffered saline (PBS)/well, sealed with 90% glycerol, and viewed using fluorescence microscopy.

### Neutralization assay

MAbs and HRIG were diluted from 10^− 1^ to 10^− 6^ in MEM-10 (MEM containing 10% FBS); 100 μl of a mixture of MAb and HRIG was transferred to six replicate wells of the 96-well plate; PBS was added to another six replicate wells, and another six replicate wells were used as a cell-only control. Then, 100 μl of the challenge street RABV at a titer of 50 FFD_50_/100 μl was added to the wells and mixed. The street RABV back titration was also performed by adding 100 μl of 50 FFD_50_/100 μl, 5 FFD_50_/100 μl, and 0.5 FFD_50_/100 μl viruses each to the six replicate wells. The plates were then incubated at 37 °C for 60 min to allow neutralization of the virus before 100 μl Neuro-2a cells at a concentration of 5 × 10^5^ cells/ml was added to all wells. The plate was incubated at 37 °C in 5% CO_2_ for 48 h, and then a DFA assay was performed to enumerate the infection foci.

### Rapid fluorescent focus inhibition test

The challenge virus standard (CVS-11) at the dose that caused 80% infection of BSR cells after 24 h was incubated with serial dilutions of the sera to be titrated. A reference serum (30 IU/ml) was included in each test. After 1 h of incubation at 37 °C, BSR cells were added to each well. After 24 h of incubation at 37 °C in 5% CO_2_, the percentage of infected cells at each serum dilution was estimated. This allowed the determination of the titer of the unknown neutralizing antibodies compared with that of the reference serum. The titer of the neutralizing antibodies in the sera was recorded as IU/ml, which is the global standard. Serum with a neutralizing antibody titer ≥0.5 IU/ml was considered positive.

### Data analysis

Statistical analysis and graphing were performed using the commercially available software GraphPad Prism 5.0 (GraphPad Software, CA, US) and Microsoft Office Excel 2007 (Microsoft Corp., Redmond, WA, US). Specifically, the intraclass correlation coefficient (ICC) and standard deviation (SD) were used to evaluate the repeatability and reliability of the method.

## Results

### Titration of street RABV

Twenty-four strains of street RABV were titrated in Neuro-2a cells, and 12 street RABV strains from 11 provinces, which showed the better multiplication capacity (> 10^5^ FFD_50_/ml, Table 2), were chosen for inclusion in the street RABV library to be used to detect the neutralizing activity of rabies virus-specific MAbs.

**Table 2:** Classification and titers of the strains included in the street RABV library

Code	Titer (FFD_50_/ml)	Host	Province
CAH0501D	3 × 10^5^	Dog	Anhui
CJS0621D	6 × 10^6^	Dog	Jiangsu
CSC1016D	5 × 10^7^	Dog	Sichuan
CJX0903D	4 × 10^6^	Dog	Jiangxi
CHN0610H	1 × 10^5^	Human	Hunan
CSX0904D	4 × 10^7^	Dog	Shanxi
CNX8601	3 × 10^5^	Human	Ningxia
CNX8511	5 × 10^7^	Human	Ningxia
CYN1009D	2 × 10^6^	Cow	Yunnan
GDZQ45	7 × 10^5^	Dog	Guangdong
CNM1103C	4 × 10^6^	Cow	Inner Mongolia
CQH1202D	3 × 10^7^	Dog	Qinghai

### Phylogenetic characteristics of the Chinese street RABVs

**Fig. 1:**
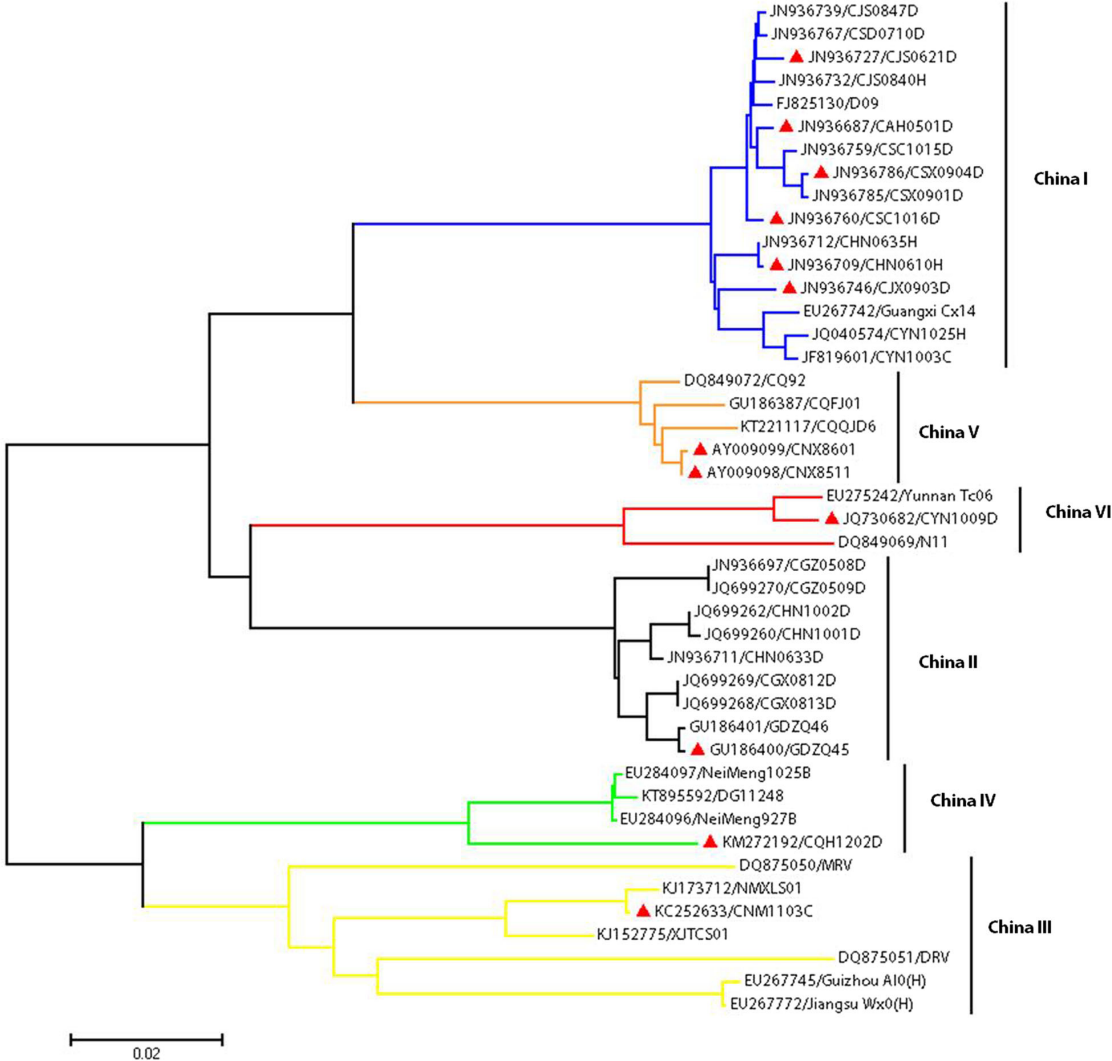
NJ phylogenetic tree of all the Chinese street RABVs classified according to the glycoprotein gene sequences. These isolates can be divided into six lineages (CHINA I–VI), which are distinguished by the different colored branches. The red triangles indicate the strains chosen for inclusion in the street RABV library. Taxa are given in the format (GenBank Accession No./strain) RABV: Rabies virus

Glycoprotein genes of 44 Chinese street RABV strains were used to construct a phylogenetic tree (Fig. 1). Of these strains, 16 belonged to the China I lineage, nine to the China II lineage, seven to the China III lineage, four to the China IV lineage, five to the China V lineage and three to the China VI lineage. The 12 strains included in the street RABV library were distributed over six lineages as follows: six strains from six different provinces belonged to the China I lineage, two strains to the China V lineage, and one strain each to the China II, China III, China IV, and China VI lineages.

### Interpretation and determination of the MAb titers against street RABVs

In the test system, the cell control wells should contain no infected cells, while the PBS positive control wells have the maximum number of foci. For the back titration of street RABV strains, the six wells containing the challenge virus dose should all be positive; the acceptable range for positive wells at the 10^− 1^ dilution was 4–6 wells and that at the 10^− 2^ dilution was 0–3 wells.

The percentage of wells containing infected cells at each dilution was calculated, and the Reed–Muench method was then used to calculate the difference between the logarithm of the starting dilution and the logarithm of the 50% endpoint dilution (difference of the logarithms) using the following formula:

$$ \frac{50\%-\left(\ \mathrm{infectivity}\ \mathrm{next}\ \mathrm{below}\ 50\%\right)}{\left(\ \mathrm{infectivity}\ \mathrm{next}\ \mathrm{above}\ 50\%\right)-\left(\ \mathrm{infectivity}\ \mathrm{next}\ \mathrm{below}\ 50\%\right)}\times \mathrm{logarithnm}\ \mathrm{of}\ \mathrm{dilution}\ \mathrm{factor} $$

Because the infectivity increases as the dilution increases, the 50% endpoint dilution is higher than the starting dilution.

Finally, the relative potency of the MAbs (IU/ml) was calculated using the following equation:

$$ \frac{50\%\mathrm{endpoint}\ \mathrm{dilution}\ \mathrm{of}\ \mathrm{MAbs}\ \mathrm{against}\ \mathrm{street}\ \mathrm{RABV}}{50\%\mathrm{endpoint}\ \mathrm{dilution}\ \mathrm{of}\ \mathrm{HRIG}\ \mathrm{against}\ \mathrm{street}\ \mathrm{RABV}}\times \mathrm{titer}\ \mathrm{of}\ \mathrm{HRIG} $$

### Repeatability of the method established for detecting the MAb neutralizing activity

**Fig. 2:**
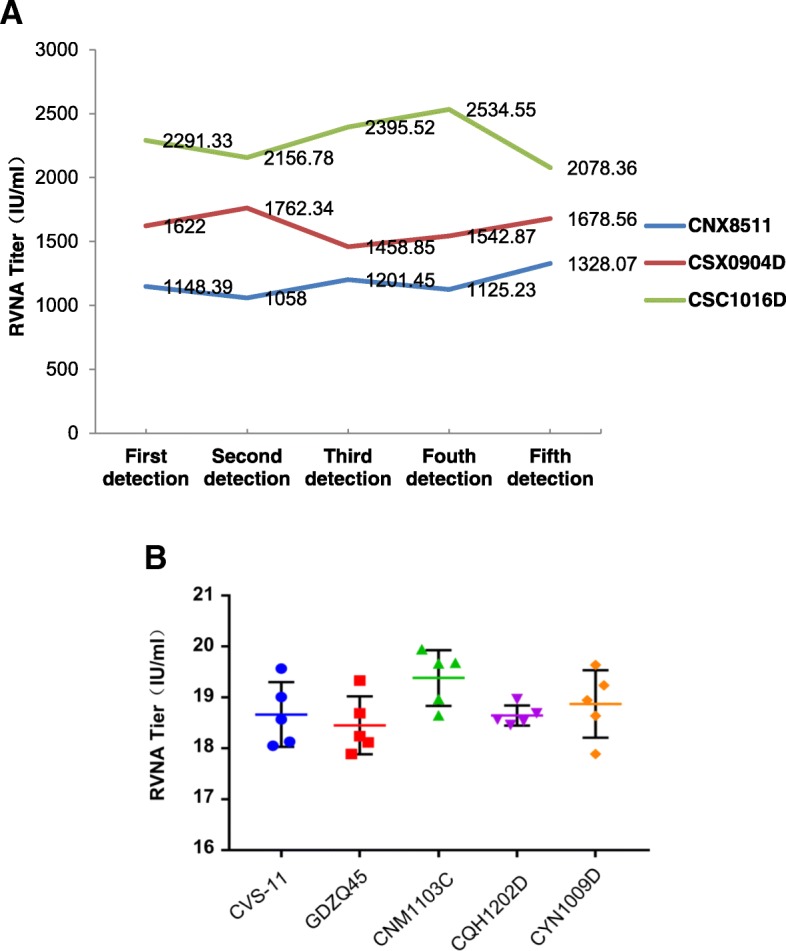
Repeatability test of the method. **a** Five replicate experiments were performed using the method, and CNX8511, CSX0904D, and CSC1016D were used as the challenge viruses. **b** Postvaccination serum was detected 5 times using RFFIT and the method that we established. GDZQ45, CNM1103C, CQH1202D and CYN1009D were used as the challenge viruses

Five replicate experiments were performed on different days to assess the repeatability and reliability of the method (Fig. 2a). Three strains of street RABVs from the library, namely, CNX8511, CSX0904D, and CSC1016D, were chosen as the challenge viruses. CNX8511 is from the China V lineage, and CSX0904D and CSC1016D are from the China I lineage. The results were analyzed using the ICC (Intraclass Correlation Coefficient) and showed that the ICC values were all > 0.92, which indicates excellent reliability.

The rapid fluorescent focus inhibition test (RFFIT) is a WHO standard assay for determining the neutralizing activity of the rabies virus in serum, which is mostly used to evaluate the immunity effect after vaccination against rabies. To compare the reliability of the method with that of RFFIT, RFFIT titers of a postvaccination serum sample were used as a control to compare with those derived from the method established in this study. In the experiment, GDZQ45, CNM1103C, CQH1202D and CYN1009D, which belonged to the China II, China III, China IV and China VI lineages, respectively, were used to determine the titers of the positive serum. The SD values of the titers obtained from the four strains of street rabies viruses were all ≤0.66, which were comparable with that of RFFIT (0.63) (Fig. 2b), suggesting no significant difference between the repeatability of the two methods. Together, the repeated measuring of the MAb or the positive serum showed the excellent reliability and stability of the method established in this study.

## Discussion

Because the WHO has strongly encouraged the development of a replacement for HRIG [5], mouse and human MAbs against the rabies virus have recently been generated. Two products are in advanced clinical trials, namely, CL184 (produced by Crucell and based on the combination of two antibodies called CR57 and CR4098) and RMAb (produced by MassBiologics and the Serum Institute of India and based on a single MAb). Moreover, these MAb products have the advantages of good specificity, high affinity, and low price, and they are easier to manufacture than the current immune globulin therapy [9–13]. Cocktails of the MAbs are promising; for instance, a combination of monoclonal antibodies (ZMapp) optimized from two previous antibody cocktails was able to rescue 100% of rhesus macaques when treatment was initiated up to 5 days post challenge [14]. An important requirement for MAbs is that they confer similar rabies virus-neutralizing activity to that of HRIG. In the selection and development of a safe and effective MAb-based PEP for rabies virus infections, it is of paramount importance to identify MAbs that are able to neutralize street RABVs from all lineages.

As part of the Chinese national surveillance program from 2004 to 2015, more than 7 919 specimens of dog brain, cow brain, human brain or saliva, and ferret-badger (*Melogale moschata*) brain were collected in 15 province/municipality/autonomous region(s) (Hunan, Guangxi, Guizhou, Jiangsu, Zhejiang, Shandong, Shanghai, Anhui, Shaanxi, Jiangxi, Sichuan, Guangdong, Qinghai, Inner Mongolia, and Yunnan) and tested for the presence of the rabies virus using the DFA test [15]. During this surveillance, more than 300 rabies virus-positive samples were collected from 23 provinces in China. Current reports indicate that there are now six lineages of street RABV in China [6, 7]. China I, II, V, and VI are sublineages of the Asian clade, China III corresponds to the Cosmopolitan clade, and China IV corresponds to the Arctic-like clade. In this study, 12 strains of street RABV with the highest titers were selected to establish a street RABV library, which covered 11 different provinces of China and six Chinese lineages (China I–VI) [16], and included six China I strains from six different provinces, two China V strains from Ningxia Province, and one strain each of China II, China III, China IV, and China VI.

This library of street RABVs can be used to evaluate the spectrum of reactivity and neutralizing capacity of any antirabies MAbs, either from China or from other countries. Moreover, the method can not only detect the neutralization activity of MAbs but also detect their titer. In addition to Mab, we also compared the reliability of the method compared with that of RFFIT. The results of our analysis showed that the method had good repeatability. Although some pseudotype virus neutralization tests have been established to test the spectrum of activity of Lyssavirus-neutralizing antibodies, these tests have the limitation that the propagation-competent viruses are easy to mutate, so the eventual evolution of resistant viruses cannot be ruled out [17]. The street RABVs used in our study are street rabies viruses that maintained their original characteristics, with the result that the neutralization assay used in our street RABV library is more stable than the assays that use pseudotype viruses.

More and more street RABV are isolated and identified as we work, we will continue to improve the library to increase the number of the street RABV library and the host, district and lineage coverage, which can be applied to the detection of neutralizing activity of Mabs and so on.

## Conclusion

In this study, we established a method for detecting the neutralizing activity of monoclonal antibodies against street rabies virus. A rabies virus library consisting of 12 strains of street RABV from 11 different provinces of China, which included six Chinese lineages (China I-China VI) and grew to high titers in N2A, was built. This study established a street RABV library reflecting the epidemiological features of Chinese rabies viruses that provides a platform for detecting the neutralizing activity of MAbs against rabies viruses.

## Additional file

Additional file 1: Multilingual abstracts in the five official working languages of the United Nations. (PDF 236 kb)

## Abbreviations

DFA: Direct fluorescent antibody
ERIG: Equine rabies immune globulin
HRIG: Human rabies immune globulin
ICC: Intraclass correlation coefficient
MAbs: Monoclonal antibodies
MEM: Minimum essential medium
PBS: Phosphate-buffered saline
PEP: Postexposure prophylaxis
RABV: Rabies virus
RFFIT: Rapid fluorescent focus inhibition test
RIG: Rabies immune globulin
WHO: World Health Organization
