# Supplementary material for: Establishment of a Chinese street rabies virus library and its application for detecting neutralizing activity
Source: Infect Dis Poverty. 2018 Dec 5;7:117. doi: 10.1186/s40249-018-0500-x (PMC6280407; doi:10.1186/s40249-018-0500-x)
Supplement: Supplementary file 1 — Multilingual abstracts in the five official working languages of the United Nations. (PDF 236 kb) [file 40249_2018_500_MOESM1_ESM.pdf]

Translation of the abstract into the five official working languages of the United Nations

إنشاء مكتبة صينية وتطبيقاتها لفيروس داء كلب الشوارع لرصد فاعلية الاستعدال

بنغ-تشنغ يو، شياو-يان تاو، لي-هوا وانغ، تشينغ تانغ، لي-يون فان، شو-شيا جانغ، شو-تشينغ ليو، شو-شين لو، غي-تشن وو، وو-يانغ جو

ملخص

معلومات عامة: لحقنة الغلوبولين المناعي ضد داء الكلب (RIG) أهمية كبرى في السيطرة على الحيوانات المشتبه بتعرضها لداء الكلب من الفئة الثالثة. بسبب تكلفة الغلوبولين المناعي ضد داء الكلب المرتفعة وتوافره المحدود، أحد البدائل المحتملة له هي الأضداد أحادية المنشأ (MAbs) ضد فيروس داء الكلب(RABV). ونتيجة لذلك، من الضروري رصد فعالية استعدال الأضداد أحادية المنشأ ضد فيروسات داء الكلب، لاسيما فيروس داء كلب الشوارع. بيد أن طريقة رصد فاعلية استعدال الأضداد أحادية المنشأ ضد فيروس كلب الشوارع مازالت مجهولة.

الطرق: لإنشاء طريقة لرصد فاعلية استعدال المضادات أحادية المنشأ ضد فيروس داء كلب الشوارع، أنشأنا مكتبة تتكون من 12 سلالة من فيروس داء كلب الشوارع من 11 مقاطعة في الصين. باستخدام مكتبة فيروس داء كلب الشوارع هذه وصيغة ريد مونش، أنشأنا طريقة للكشف عن عيار استعدال الأضداد أحادية المنشأ. تم تقييم ثبات وتكرار هذه الطريقة بالقياس المتكرر لفاعلية استعدال الأضداد أحادية المنشأ وبمصل بعد التطعيم.

النتائج: تم اختيار ما مجموعه12 سلالة من فيروس داء كلب الشوارع لتضمينها في مكتبة فيروس داء كلب الشوارع، التي غطت ستة سلالات صينية (الصين 1 إلى الصين 6) ونمت إلى معايير أعلى في خلايا N2A ($> 10^5$ FFD_{50}/ ml). واستناداً إلى المكتبة، أنشأنا طريقة الكشف عن فاعلية استعدال الأضداد أحادية المنشأ. وأظهرت نتائج قياس الأضداد أحادية المنشأ المتكررة والمصل الإيجابي، موثوقية وقابلية تكرار ممتازة للطريقة المحددة في هذه الدراسة.

الاستنتاجات: أنشأت هذه الدراسة لأول مرة مكتبة فيروس داء كلب الشوارع التي تعكس السمات الوبائية لفيروسات داء الكلب في الصين، وتوفر منصة للكشف عن فاعلية استعدال الأضداد أحادية المنشأ ضد فيروسات داء الكلب المنتشرة في الصين.

Translated from English version into Arabic by Nirmin Hashoum and Amal Jamal, through

TRANSLATORS WITHOUT BORDERS

中国狂犬病街毒文库的建立及其在中和抗体活性检测中的应用

于鹏程，陶晓燕，王力华，唐青，范丽云，张曙霞，刘淑清，卢学新，武桂珍，朱武洋

摘要

引言：狂犬病免疫球蛋白（RIG）的使用对于 III 级暴露后的处置至关重要。RIG 的生产成本高且数量有限，因此针对狂犬病病毒（RABV）的单克隆抗体（MAb）是代替 RIG 的可行途径。检测 MAb 针对狂犬病病毒，尤其是针对狂犬病街毒株的中和活性非常必要。然而，目前还没有建立 MAb 针对狂犬病街毒株的中和活性检测方法。

方法：为了检测狂犬病病毒单克隆抗体针对街毒株的中和活性，我们构建了由中国 11 个省份的 12 株狂犬病病毒组成的街毒文库。使用这个街毒文库和 Reed-Muench 公式，建立了 MAb 中和活性的检测方法。最后通过重复检测 MAb 和免疫后血清的中和活性来评价该方法的可靠性和稳定性。

结果： 共有 12 株狂犬病街毒株入选文库，分别属于 6 个中国谱系（China I-China VI），在

N2A 细胞上具有良好的感染力（>10^5 FFD$_{50}$/ml ）。在该街毒文库的基础上，我们构建了 MAb 中和活性的检测方法，经过反复检测 MAb 和免疫后血清的中和活性，结果显示该方法具有良好的可靠性和稳定性。
结论：本研究建立了具有中国狂犬病病毒流行特征的 RABV 街毒文库，为检测狂犬病病毒 MAb 针对中国流行的狂犬病街毒的中和活性提供了平台。

Translated from English version into Chinese by Jin Chen, edited by Pin Yang

Élaboration d'une libraire du virus de la rage de rues en Chine et ses applications pour détecter une activité neutralisante

Peng-Cheng Yu，Xiao-Yan Tao，Li-Hua Wang，Qing Tang，Li-Yun Fan, Shu-Xia Zhang, Shu-Qing Liu，Xue-Xin Lu, Gui-Zhen Wu, Wu-Yang Zhu

Résumé

Contexte : L'injection d'immunoglobulines antirabiques (RIG) est de la plus grande importance dans la gestion des expositions de catégorie III aux animaux suspectés d'avoir la rage. En raison du coût élevé et de la faible disponibilité des RIG existantes, les anticorps monoclonaux (MAbs) représentent un remplacement possible contre le virus de la rage (RABV). Par conséquent, il est nécessaire de déterminer l'activité neutralisante des MAbs contre les virus de la rage, tout particulièrement le virus de la rage des rues. Cependant, la méthode visant à détecter l'activité neutralisante des MAbscontrele virus de la rage des ruesreste indéfinie.
Méthodes : Pour établir une méthode visant à détecter l'activité neutralisante des MAbs contre le virus de la rage des rues, nous avons construit une librairie comprenant 12 souches de RABV des rues provenant de 11 provinces en Chine. En utilisant cette bibliothèque RABV de rue et la formule de Reed – Muench, nous avons mis au point une méthode de détection du titre neutralisant des MAbs. La fiabilité et la répétabilité de la méthode ont été évaluées en mesurant à maintes reprises l'activité de neutralisation d'un MAb et d'un sérum après la vaccination.
Résultats : Un total de 12 souches de RABV des rues ont été choisies pour faire partie de lalibrairie de RABV des rues qui couvrent six lignages chinois (Chine I–Chine VI) et ont produit des concentrations élevées dans les cellules N2A (>10^5 FFD$_{50}$/ml). Sur la base de la librairie, nous avons construit la méthode visant à détecter l'activité neutralisante des MAbs. Les résultats des mesures répétées des MAbs et sérums positifs ont démontré une excellente fiabilité et répétabilitéde la méthode établie dans cette étude.
Conclusions : Cette étude a établi pour la première fois une librairie de RABV des rues reflétant les caractéristiques épidémiologiques des virus des rages chinoises, ce qui fournit une plateforme pour détecter l'activité de neutralisation des MAbs contre les virus de la rage circulant en Chine.

Translated from English version into French by Cathy Davies and Akshah Nathan, through

Создание библиотеки китайских вирусов бешенства среди уличных животных и её использование для нахождения нейтрализующей активности

Пэн-Чэн Юй, Сяо-Янь Тао, Ли-Хуа Ван, Цин Тан, Ли-Юнь Фань, Шу-Ся Чжан, Шу-Цин Лю, Сюэ-Синь Лу, Гуй-Чжэнь У, У-Янь Чжу

Аннотация

История вопроса: Инъекция антирабического иммуноглобулина (АИ) чрезвычайно важна при управлении контактами третьей категории с животными с подозрением на бешенство. Вследствие высокой стоимости и ограниченной доступности АИ в качестве возможной замены могут служить моноклональные антитела (мкАТ) к вирусу бешенства (ВБ). Следовательно, необходимо определить нейтрализующую активность мкАТ против вирусов бешенства, особенно у уличных животных. Однако метод распознавания нейтрализующей активности мкАТ против вируса бешенства уличных животных до сих пор остаётся не определённым.

Методы: Для определения метода распознания нейтрализующей активности мкАТ против вируса бешенства у уличных животных мы создали библиотеку, состоящую из12 штаммов вируса, собранных в 11 провинциях Китая. С помощью этой библиотеки ВБ и формулы Рида-Менча мы разработали способ распознавания нейтрализующего титра мкАТ. Надежность и воспроизводимость этого метода оценивались путем повторных замеров нейтрализующей активности мкАТ и поствакцинальной сыворотки.

Результаты: В общей сложности 12 штаммов ВБ были включены в библиотеку ВБ по уличным животным, в которую вошли шесть китайских линий (Китай I – Китай VI) и достигли высоких титров в клетках N2A (>10^5 FFD_{50}/мл). На основании данной библиотеки мы разработали метод распознания нейтрализующей активности мкАТ. Результаты повторных замеров мкАТ и положительная сыворотка доказали высокую надежность и воспроизводимость метода, разработанного при данном исследовании.

Выводы: В данном исследовании впервые была создана библиотека ВБ по уличным животным, отражающая эпидеомологические особенности китайских ВБ, что стало основой для распознания нейтрализующей активности мкАТ против распространённых в Китае вирусов бешенства.

Translated from English version into Russian by Elena McDonnell and Liudmila Tomanek, through

Creación de una biblioteca del virus "calle" de la rabia en China y su uso para detectar actividad neutralizante

Peng-Cheng Yu，Xiao-Yan Tao，Li-Hua Wang，Qing Tang，Li-Yun Fan, Shu-Xia Zhang, Shu-Qing Liu，Xue-Xin Lu, Gui-Zhen Wu, Wu-Yang Zhu

Resumen

Antecedentes: La inyección de inmunoglobulina antirrábica humana (IGRH) es el paso de mayor importancia en el tratamiento de exposiciones de categoría III a animales que puedan ser portadores de rabia. Debido al alto coste y a la limitada disponibilidad de existencias de IGRH, un posible sustituto para la IGRH contra el virus de la rabia (RABV) son los anticuerpos monoclonales (MAbs). En consecuencia, es necesario determinar la actividad neutralizante de los MAbs contra el virus de la rabia, especialmente contra el virus "calle". No obstante, aún se desconoce el método para detectar la actividad neutralizante de los MAbs contra el virus "calle" de la rabia.

Metodología: Para establecer un método de detección de la actividad neutralizante de las MAbs contra el virus "calle", hemos creado una biblioteca con12 cepas del virus "calle" RABV de 11 provincias chinas. Gracias a esta biblioteca y la fórmula de Reed y Muench, hemos establecido un método para determinar el título neutralizante de los MAbs. Asimismo, hemos evaluado la fiabilidad y repetibilidad del método mediante la medición reiterada de la actividad neutralizante de un MAb y de un suero post vacuna.

Resultados: Seleccionamos 12 cepas del virus "calle"RABV para incluirlas en la biblioteca que corresponden a seis clanes chinos (China I – China VI) y alcanzaron altos títulos de células N2A ($>10^5$ FFD_{50}/ml). A partir de esta biblioteca, hemos diseñado el método para detectar la actividad neutralizante de los MAbs. Los resultados de la medición reiterada de los MAbs y suero positivo mostraron una excelente fiabilidad y repetibilidad del método aplicado en el estudio.

Conclusiones: Este estudio establece por primera vez una biblioteca del virus calle RABV que refleja las características epidemiológicas del virus de la rabia chino, lo que proporciona una plataforma para detectar la actividad neutralizante de los MAbs contra las cepas que circulan por China.

Translated from English version into Spanish by David López Canelada and Clarines, through

TRANSLATORS
WITHOUT BORDERS
